# Role of mGlu5 in Persistent Forms of Hippocampal Synaptic Plasticity and the Encoding of Spatial Experience

**DOI:** 10.3390/cells11213352

**Published:** 2022-10-24

**Authors:** Hardy Hagena, Denise Manahan-Vaughan

**Affiliations:** Department of Neurophysiology, Medical Faculty, Ruhr University Bochum, 44780 Bochum, Germany

**Keywords:** mGlu5, plasticity, hippocampus, spatial learning, long-term memory, positive allosteric modulators, negative allosteric modulators

## Abstract

The metabotropic glutamate (mGlu) receptor family consists of group I receptors (mGlu1 and mGlu5) that are positively coupled to phospholipase-C and group II (mGlu2 and mGlu3) and III receptors (mGlu4-8) that are negatively coupled to adenylyl cyclase. Of these, mGlu5 has emerged as a key factor in the induction and maintenance of persistent (>24 h) forms of hippocampal synaptic plasticity. Studies in freely behaving rodents have revealed that mGlu5 plays a pivotal role in the stabilisation of hippocampal long-term potentiation (LTP) and long-term depression (LTD) that are tightly associated with the acquisition and retention of knowledge about spatial experience. In this review article we shall address the state of the art in terms of the role of mGlu5 in forms of hippocampal synaptic plasticity related to experience-dependent information storage and present evidence that normal mGlu5 function is central to these processes.

## 1. Introduction

Metabotropic glutamate (mGlu) receptors comprise a family of G-protein coupled receptors that are present throughout the brain [1,2] and are highly expressed in the hippocampus [2]. They are specifically involved in the regulation of persistent (>24 h) forms of hippocampal synaptic plasticity, such as long-term potentiation (LTP) and long-term depression (LTD) [3,4,5,6,7,8], hippocampus-dependent learning [9,10,11,12,13,14,15].They also support hippocampal information transfer by means of neuronal oscillations [16,17] and the beneficial effects of environmental enrichment on hippocampal function [18].

Glutamate is the most abundant/primary neurotransmitter in the central nervous system and exerts its action by binding to ionotropic (iGlu) and metabotropic (mGlu) glutamate receptors [19,20,21,22,23]. In contrast to iGlu receptors, which are ligand-gated ion-channels, activation of mGlu receptors initiates G-protein-coupled signaling cascades. The mGlu receptors are subdivided into three groups, based on their biochemical coupling and signaling cascades [24].

Group I mGlu receptors, comprising mGlu1 and mGlu5 [25,26], are particularly important for enabling forms of hippocampal synaptic plasticity that persist for very long periods (>24 h) [27]. Group II (mGlu2 and 3), and group III mGlu receptors (mGlu 4–8) are negatively coupled to adenylyl cyclase [24] and mostly function as autoreceptors for glutamate [28,29,30,31,32], serving, for example, to lower excitability levels in the hippocampus [33]. Activation of these receptors raises the threshold for the induction of synaptic plasticity, or favours changes in synaptic strength that promote LTD [34,35,36,37].

Another differentiation of the separate mGlu receptor groups can be made based on their location of expression in the brain. Group I mGlu receptors are mainly expressed on postsynaptic sites [38,39,40], whereas group II and III mGlu receptors are mainly expressed presynaptically [41]. The expression of mGlu5 receptors is, correspondingly, mostly located postsynaptically and the highest expression can be found in the CA1 subregion of the hippocampus, whereas comparatively less expression of this receptor has been found in the dentate gyrus (DG) and the CA3 region [2,41].

Of the abovementioned family of mGlu receptors, mGlu5 stands out as being of particular importance for information processing and storage in the hippocampus relating to the acquisition and retention of long-term memory [27]. Furthermore, mGlu5 contributes to homeostatic brain function by means of the fine-tuning of synaptic plasticity [42]. For this reason, it is perhaps unsurprising that dysfunction of mGlu5 receptors is associated with several neurological diseases that impact specifically on hippocampal function, such as epilepsy [43,44,45], psychosis [46,47,48,49,50,51], Alzheimer’s disease (AD) [52,53,54,55,56,57,58,59], Parkinson’s disease [60,61,62,63], Fragile X [64,65,66,67,68,69,70,71,72] and Rett Syndrome [73,74,75,76]. In fact, it has also been proposed that mGlu5 may be directly involved in the pathophysiology of these diseases. For example, the propagation of epileptiform activity by N-methyl-D-aspartate receptors (NMDAR) is supported by astrocytic mGlu5 receptor activation [77], and in AD, Aβ oligomers cause a shift of mGlu5 receptors towards the synapse, a process that may promote synaptotoxicity [78]. MGlu5 receptors also support the coupling of soluble amyloid-β oligomers and cellular prion protein [79] and, thus, directly impact synaptic plasticity. For example, it was reported that an mGlu5 receptor negative allosteric modulator rescued Aβ_1-42_-induced inhibition of LTP and prevented the induction of LTD in Aβ_1-42_ treated animals [80]. In Fragile X and Rett Syndrome, mGlu5 receptors directly interact with Fragile X mental retardation protein (FMRP) [64,75,78]. In light of these discoveries, mGlu5 receptors have been subjected to scrutiny as a promising target for the treatment of such diseases [53,57,81,82,83,84,85,86].

## 2. Contribution of mGlu5 to Persistent Forms of Hippocampal Synaptic Plasticity

Persistent synaptic plasticity refers to long-lasting (>24 h) changes in synaptic transmission and is expressed in the form of LTP and LTD, both of which facilitate hippocampus-dependent spatial memory [87,88,89,90,91,92,93]. For this reason, these processes are typically investigated in freely behaving rats and mice [93]. MGlu5 is positively coupled to phospholipase C and activation of the receptor leads to the generation of inositol trisphospate (IP_3_) [24,94,95]. The binding of IP_3_ to receptors on the endoplasmic reticulum leads to release of calcium into the cytosol and subsequent activation of enzymes, such as protein kinase C (PKC), that are stimulated by intracellular Ca^2+^ elevations [96,97,98,99]. This supports a whole variety of biochemical signaling cascades that are conducive towards the maintenance of LTP and LTD for periods of hours and more [100,101,102,103,104,105,106,107,108,109]. For example, an increase in phosphorylation of protein F1, a substrate PKC, is directly related to long-term changes in synaptic enhancement [110]. Furthermore, activation of PKC results in the phosphorylation of the serine 818 residue of GluR1 (GluA1), a critical step that is required for LTP [103].

### 2.1. Contribution of mGlu5 to Long-Term Potentiation

In the hippocampus, LTP is typically induced in vivo via activation of NMDARs [111,112,113,114,115,116,117,118,119,120] although NMDAR-independent forms have also been described in behaving rodents [121,122]. Numerous studies have also described the necessity of mGlu5 receptors for the induction and maintenance of LTP in rodents in vivo [11,18,27,34,123,124]. Furthermore, activation of mGlu5 receptors, either with an agonist [125,126] or positive allosteric modulator (PAM), results in the enhancement of LTP [127,128,129,130]. It should be mentioned that not all hippocampal subregions display the same dependency of persistent LTP on mGlu5 receptors. Whereas perforant path (pp)–DG, mossy fiber (MF)–CA3 and Schaffer collateral (SC)–CA1 synapses require activation of mGlu5 to express LTP (>24 h) [3,5,11], associational commissural–CA3 synapses do not [3].

Following the induction of LTP, a first phase, called early-LTP (E-LTP, or short-term potentiation, STP) that lasts 2–3 h can be distinguished from late LTP (L-LTP) [131,132,133,134]. It is E-LTP/STP that typically depends on the activation of NMDARs [135,136,137,138]. The importance of mGlu5 receptors in this process has been shown in mGlu5 knock-out mice, where the NMDAR component of LTP is abolished [139]. In freely behaving rats, pharmacological antagonism of mGlu5 receptors dose-dependently impairs STP [11]. Mechanistically, mGlu5 receptor activation serves to potentiate NMDAR currents [140,141,142,143], a process that is dependent on intracellular Ca^2+^-release and PKC-activation [139,143,144].

NMDARs that support synaptic plasticity in the hippocampus are composed of GluN1, GluN2A and GluN2B subunits [145]. Whether glutamate binding to NMDARs results in LTP or LTD depends on the pattern of afferent stimulation [111,117], as well as on the subunit composition of the NMDAR, which switches from a high to low GluN2B/GluN2A ratio during development [146,147,148]. This change in ratio is dependent on activation of mGlu5 receptors. MGlu5 receptor knock-out mice, for example, show a deficiency in the activity-dependent switch from GluN2B to GluN2A in the hippocampus and visual cortex, and application of an mGlu5 receptor antagonist inhibits the sensitivity of EPSCs initiated by application of the GluN2B inhibitor ifenprodil [149]. The contribution of GluN2A and/or GluN2B to LTP and LTD depends on the frequency of postsynaptic depolarisation [150,151], as well as the pattern of impulses delivered by the afferent input [111]. Furthermore, the threshold for induction of GluN2B-dependent hippocampal LTD is lowered when mGlu5 is activated [152], and transgenic mice that lack mGlu5 fail to express NMDAR-dependent hippocampal LTP [139]. Thus, alterations in mGlu5 receptor function may alter the direction of change in frequency-dependent hippocampal synaptic plasticity, or even serve to hinder the induction of a specific form of synaptic plasticity.

**Figure 1:**
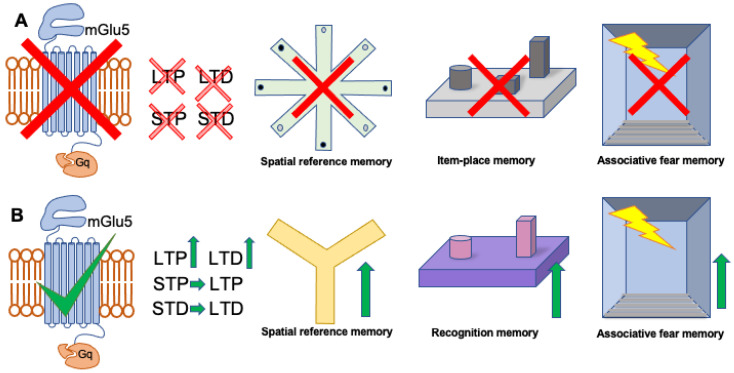
Schema of the effects of pharmacological antagonism, or agonism, of mGlu5 receptors on hippocampal synaptic plasticity and hippocampus-dependent forms of learning and memory. (**A**) Pharmacological antagonism of mGlu5 receptors prevents the maintenance of both LTP and LTD. STP and STD are also impaired. Prior treatment with an mGlu5 receptor antagonist prevents spatial reference memory in paradigms such as the 8-arm radial maze, prevents effective item-place memory and disrupts associative fear memory. (**B**) Pharmacological activation of mGlu5 receptors strengthens and prolongs LTP and LTD and also facilitates short-term plasticity (STP, STD) into long-term plasticity (LTP, LTD respectively). Spatial learning in paradigms such as the Y-maze is enhanced, recognition memory is improved, as is associative fear memory. For relevant literature, see Table 1 and Table 2. The red cross signifies inhibition/impairment. The green tick signifies activation, and the green arrows signify enhancement.

The necessity of mGlu5 receptor activation for persistent forms of LTP has been described for perforant path–dentate gyrus, Schaffer collateral–CA1 and mossy fiber–CA3 synapses in mouse and rat hippocampi (Table 1, Figure 1) [3,5,11,153]. Sustained changes in synaptic efficacy (i.e., longer than 24 h) require protein synthesis [154,155,156], typically mediated by activation of immediate early genes (IEGs). Many signaling cascades that eventually lead to the activation of IEGs are dependent on mGlu5 receptor activation. For example, activation of Ca2+/calmodulin-dependent protein kinase (CaMK) and the phosphorylation of ERK1/2 are crucial steps in the activation of the c-fos IEG [157], processes that are supported by activation of mGlu5 receptors [158]. Furthermore, mGlu5 receptor activation is involved in the modulation of other downstream targets, such as binding to the IEG, Homer 1a [159,160]. The interaction of Homer and Shank, two proteins of the postsynaptic density (PSD), result in morphological changes in dendritic spines [161] and to an association of group I mGlu receptors with the NMDAR signaling pathway [162,163]. Furthermore, increased levels of mGlu5 receptors and Homer1 proteins in Sin3aNH transgenic mice result in enhanced hippocampus-dependent memory and synaptic plasticity [164]. Another element in the mGlu5 receptor mediation of synaptic plasticity involves their interaction with PSD-95/Disk large/ZO-1 (PDZ) domains [165]. Tamalin, a PDZ domain-containing protein, is crucial for trafficking and cell surface expression of mGlu5 receptors [166]. Preventing the interaction of group I mGlu receptors and Tamalin impairs the expression of LTD, but not LTP, in the presence of an mGlu5 receptor agonist [7]. These results support the crucial role of PDZ-domain proteins in mGlu5 receptor mediated hippocampal LTD. Thus, the mechanistic regulation by the mGlu5 receptor of hippocampal synaptic plasticity extends beyond its biochemical coupling to phospholipase C and regulation of NMDAR function, but it is also enabled by its modulation of a variety of intracellular targets. The modulation by mGlu5 receptors of cell surface and intracellular targets related to the induction and maintenance of LTP, suggest, in turn, that perturbation of mGlu5 receptor activity will have profound effects on this form of persistent synaptic plasticity. This has been shown in a variety of studies using pharmacological antagonism, or transgenic deletion of mGlu5 receptors [3,8,11,16,127,153,167].

### 2.2. Contribution of mGlu5 Receptors to Long-Term Depression

Hippocampal LTD is also significantly regulated by mGlu5. Initial studies reported that LTD could be induced by agonist activation of mGlu5 in hippocampal slice preparations [172]. Later, in vivo studies showed a more complex regulation of input-specific, experience-dependent LTD by mGlu5 receptors in the hippocampus of freely behaving rats and mice (Table 1, Figure 1, [3,4,5,123,169]). In this case, LTD was induced by patterned stimulation of afferent fibres within the hippocampus and the effects of pharmacological agonism, or antagonism of mGlu5 receptors was explored. Here, effects differ depending on the hippocampal subregion. For example, at pp–DG, SC–CA1 and AC–CA3 synapses, LTD depends on the activation of mGlu5 receptors. In contrast, LTD at MF–CA3 synapses does not require activation of this receptor [3]. Furthermore, hippocampal short-term depression (STD) is facilitated into persistent LTD by agonist activation of the receptors [123].

Interestingly, pharmacological antagonism of mGlu5 receptors prevents the induction, but not the persistency of LTD induced by high frequency stimulation of the perforant path both in vitro [168] and in vivo [5]. MGlu5 receptor antagonism results in a suppression of NMDAR currents and, thus, an inhibition of LTD [173]. Furthermore, it has been shown that mGlu5 knock-out mice fail to express LTD [172] and application of mGlu5 receptor antagonists impair both NMDAR-dependent [5] and independent forms of LTD [172,174,175,176,177]. In contrast, agonist activation of mGlu5 promotes the expression of persistent hippocampal LTD [3,4,7,169], a process that is likely to involve facilitation of currents through NMDARs that contain GluN2B [152].

## 3. Contribution of mGlu5 Receptors to Forms of Hippocampal Synaptic Plasticity That Are Enabled by Spatial Experience

The hippocampus is an essential brain structure for the processing and encoding of spatial and associative representations of experience by means of long-term synaptic plasticity [87,93,178]. LTP and LTD are the cellular mechanisms that enable the storage of this kind of information [88,89,90,93,179,180]. Numerous studies have described the specific kinds of spatial information encoded by LTP and LTD, whereby a differentiation of the relative elements of spatial memory enabled by LTP and LTD has become evident. For example, LTP is expressed in response to the de novo acquisition of knowledge about a novel spatial environment [88,93,180,181,182,183,184,185], whereas LTD is facilitated upon the acquisition, or updating, of knowledge about discrete content features of an environment [4,88,89,90,180,186,187,188,189]. All hippocampal subfields reportedly show the same LTP-specific stimulus response related to novel spatial learning [88,180,186]. The facilitation of LTD in distinct hippocampal subfields and synaptic subcompartments, on the other hand, is triggered by different kinds of spatial content information. For example, whereas Schaffer collateral–CA1 and associational commissural–CA3 synapses respond, with LTD, to novel or changed configurations of discretely located spatial items [180,186], exposure of rodents to novel, or updated, configurations of highly visible landmark objects facilitates LTD at perforant path–DG and mossy fiber–CA3 synapses [180,186]. It is not only visuospatial cues that result in the expression of LTD, however, but spatial cues generated by odours, or sound, also facilitate synaptic plasticity and thus, enable the formation of spatial memories [169,190]. This suggests that, for the hippocampus, all sensory modalities may be able to generate salient sensory information that is integrated into associative records of experience by LTP, and spatial content representations by means of LTD. In other words, LTP creates representation templates that are refined and updated by LTD [191].

Numerous studies have shown that synaptic plasticity that occurs by spatial experience depends on the activation of mGlu5 receptors, that potentiating mGlu5 receptor function results in improved learning performance in spatial tasks and that antagonism of mGlu5 receptors decreases performance in spatial learning and working or reference memory (Table 2, Figure 1). The procurement of spatial content information, such as the acquisition, or updating, of knowledge about novel object-place configurations, or of spatial object recognition, is prevented in the hippocampal CA1 region during pharmacological antagonism of mGlu5 receptors [4,5]. LTD that is facilitated by this kind of learning experience is also inhibited [4,5]. Furthermore, a tight correlation between activation of mGlu5 receptors, successful induction of LTP and learning has been shown [9,11,16,124]. These results suggest that mGlu5 receptors are crucial for the expression of hippocampal LTD and LTP, as well as for the underlying acquisition of spatial information [4,5,11,192,193,194].

**Table 2:** Summary of mGlu5 receptor contribution to hippocampal to hippocampus-dependent learningKO: knock-out; MWM: Morris water maze; NAM: negative allosteric modulators; PAM: positive allosteric modulators.

Hippocampus-Dependent Learning Task	Species	Outcome	References
**Antagonist/NAM**			
object-place configuration	rat	LTD and memory inhibited	[5]
acquisition of novel environment	rat	impaired place field stability	[17]
acquisition of novel audiospatial cues	rat	LTD inhibited	[169]
eight-arm radial maze	rat	reference and working memory impaired	[11,16,124]
four-arm plus maze	rat	impairment of spontaneous alternation behaviour	[195]
Y-Maze spatial alternation task	rat	impairment of retention; no effect if antagonist applied immediately after training	[10]
T-Maze	rat	extinction of consolidated context impaired	[196]
working and reference memory	rat	impaired performance	[124]
inhibitory avoidance learning	rat	impairment in retention	[197]
extinction learning	rat	impaired extinction of consolidated information	[196]
fear conditioning	rat	impaired expression of contextual fear conditioning	[198]
fear conditioning	mouse	attenuation of cue-elicited freezing during fear conditioning	[199]
spatial object recognition	mouse	LTD and learning inhibited	[4]
environmental enrichment (EE)	mouse	impairment of EE-mediated LTP	[18]
**Agonist/PAM**			
object recognition	rat	enhancement with low concentration of PAM	[200]
Y-maze spatial alternation task	rat	improvement in spatial alternation retention	[201]
T-maze	rat	enhanced memory abilities	[202]
MWM	mouse	enhanced learning and memory performance	[127]
MWM	mouse	impaired spatial learning	[203]
MWM	mouse	enhanced reversal learning	[204]
Barnes maze	mouse	improved performance during reversal training	[202]
fear extinction	mouse	enhanced fear extinction learning	
**mGlu5 KO**			
MWM	mouse	impaired spatial learning	[153]
fear-conditioning	mouse	impaired processing of contextual information	[153]

Novel, or constantly changing spatial and sensory stimuli within an environment, referred to as environmental enrichment, may help in preventing cognitive decline [205,206]. Environmental enrichment has also been reported to significantly improve hippocampal synaptic responses [207]. Interestingly, antagonism of mGlu5 receptors prevents enrichment-mediated improvement in synaptic plasticity, and environmental enrichment enhances mGlu5–Homer1a interactions [18,208], supporting a significant role for mGlu5 in the optimisation of cognitive performance. This possibility is, furthermore, supported by studies that show an upregulation of mGlu5 receptor expression after induction of synaptic plasticity, or hippocampus-dependent learning tasks [209,210]. As mentioned earlier, the hippocampus supports memory formation by integrating information from different sensory modalities and promoting learning-dependent synaptic plasticity that occurs in conjunction with this learning process [93,211]. These modalities comprise, for example, vision, hearing or olfaction [169,190,212,213,214,215] and all of these contribute to environmental enrichment. Here, also, mGlu5 receptors play a role. For example, receptor antagonism prevents the facilitation of hippocampal LTD that occurs in response to the novel exposure of freely behaving rats to audiospatial information [169].

The formation of a memory does not only depend on the expression of long-term synaptic plasticity but also on the creation of stable place fields [216,217] and the unhampered activity of network oscillations [171,178]. Here, too, mGlu5 receptors enable these mechanisms. Encoding of spatial memory by means of alterations in synaptic plasticity is supported by neuronal oscillations [218,219,220]. Oscillations in the hippocampus occur at theta (5–50 Hz) and gamma (30–100 Hz) frequencies, and their coupling supports the acquisition and retrieval of spatial information by means of synaptic potentiation [221,222]. Strikingly, mGlu5 receptors affect theta and gamma power: prolonged antagonism of mGlu5 receptors results in a suppression of theta and gamma activity in the dentate gyrus and subsequently leads to an inhibition of LTD [16,130]. In line with this, positive modulation with a PAM enhances LTP and leads to an increase in relative spectral and gamma power that precedes LTP [130]. Another facet of efficient cognitive performance comprises the suppression of memories and, specifically, of behaviors, that are no longer salient. The underlying process is termed extinction learning [223,224]. Here, too, mGlu5 plays a role, whereby receptor antagonism shifts extinction learning towards context-independent elements of spatial experience [196].

Spatial learning is also used as a behavioral assay to understand the mechanisms underlying the pathophysiology of brain disease and disorders. In animal models of AD, performance in different spatial learning related tasks, such as the Barnes maze [225,226] or the water maze [227] is affected. Equivalent deficits in spatial learning can be found in transgenic models of Rett syndrome [228,229,230], Fragile X [231,232] and psychosis [233,234,235]. Strikingly, a dysfunction of mGlu5 receptors has been proposed to play a crucial role in these diseases. Furthermore, PAMs acting on mGlu5 receptors can reverse cognitive deficits and, thus, are under scrutiny as potential treatments for a variety of diseases involving learning-related disorders [127,200,236].

## 4. Conclusions

In summary, studies conducted in recent decades provide evidence for a key role for the mGlu5 receptor in the successful expression of persistent synaptic plasticity that, in turn, enables the effective acquisition and retention of spatial memory. Aberrations in mGlu5 receptor functionality, thus, affect not only LTP and LTD, but also cellular mechanisms that enable the formation of spatial representations and memories, both in humans and rodents [93,237,238]. Aspects that still remain unclear are, for example, the putative role of heteromeric mGlu receptors in hippocampal synaptic plasticity and experience-dependent information encoding. Chemogenetic approaches that allow the targeting of discrete populations of mGlu5 receptors should also help decipher the role of mGlu5 receptors in local networks that support cognition. Given the prominent role of mGlu5 receptors in a variety of diseases, this receptor holds promise as a potential target for pharmaceutical intervention. Although a vast amount of pre-clinical and basic research has been conducted to evaluate the function of mGlu5 receptors, the gap to clinical applications is still large [70,239,240] and see also: https://www.fraxa.org/fragile-x-clinical-trials-mglur-theory (accessed on 28 August 2022). Future research could help bridge this gap by dissecting the molecular mechanisms downstream of mGlu5 receptor activation, with the goal of understanding how these receptors contribute to diseases and, ultimately, of overcoming the challenge of transferring knowledge gained through basic research into clinically effective strategies.

## Figures and Tables

**Table 1:** Summary of mGlu5 receptor contribution to short-term potentiation (STP), long-term potentiation (LTP), short-term depression (STD) and long-term depression (LTD) in the hippocampal perforant path–dentate gyrus (DG), mossy fiber–CA3 (MF-CA3), associational/commissural–CA3 (AC–CA3) or Schaffer collateral–CA1 (CA1) synapses in vivo and in vitro in rats or mice. KO: knock-out; NAM: negative allosteric modulators; PAM: positive allosteric modulators.

Preparation	Species	Outcome	Hippocampal Region	References
**mGlu5 antagonist/NAM**				
in vivo	rat	LTP inhibited, STP impaired	DG	[10,11,124]
in vivo	rat	L-LTP inhibited	DG	[16,130]
in vivo	rat	No effect on LTD	DG	[168]
in vivo	rat	LTP inhibited	MF–CA3	[3]
in vivo	rat	No effect on LTP	AC–CA3	[3]
in vivo	rat	No effect on LTD	MF–CA3	[3]
in vivo	rat	LTD inhibited	AC–CA3	[3]
in vivo	rat	LTP inhibited	CA1	[124]
in vivo	rat	LTP enhanced (after prolonged antagonism)	CA1	[16]
in vivo	rat	LTD inhibited	CA1	[5,33,169]
in vitro	rat	STP inhibited	CA1	[123]
in vitro	rat	L-LTP inhibited	CA1	[123]
in vitro	rat	LTD induction inhibited	CA1	[123]
in vitro	rat	L-LTD inhibited	CA1	[123]
in vitro	mouse	LTP inhibited	CA1	[170]
in vivo	mouse	STD, LTD inhibited	CA1	[4,11]
in vivo	mouse (3–4 months old)	LTP inhibited	CA1	[18]
in vivo	mouse (10–14 months old)	No effect	CA1	[18]
**mGlu5 agonist/PAM**				
in vivo	rat	LTP enhanced	DG	[130]
in vitro, hippocampal slice preparation	rat	LTP enhanced	CA1	[8,127]
in vitro, hippocampal slice preparation	rat	LTD enhanced	CA1	[127]
in vitro, hippocampal slice preparation	mouse	LTP enhanced	CA1	[8]
in vitro, hippocampal slice preparation	mouse	LTP rescued	CA1	[171]
**mGlu5 KO**				
in vitro, hippocampal slice preparation	mouse	LTP inhibited	CA1, DG	[153]
in vitro, hippocampal slice preparation	mouse	No effect	MF–CA3	[153]
in vitro, hippocampal slice preparation	mouse	DHPG-LTD inhibited	CA1	[172]

## Data Availability

Not applicable.
